# Simultaneous measurement of multiple organic tracers in fine aerosols from biomass burning and fungal spores by HPLC-MS/MS

**DOI:** 10.1039/c8ra04991b

**Published:** 2018-10-04

**Authors:** Jingsha Xu, Jun He, Honghui Xu, Dongsheng Ji, Colin Snape, Huan Yu, Chunrong Jia, Chengjun Wang, Jianfa Gao

**Affiliations:** Department of Chemical and Environmental Engineering, International Doctoral Innovation Centre, University of Nottingham Ningbo China Ningbo P. R. China; Zhejiang Meteorological Science Institute Hangzhou P. R. China; State Key Laboratory of Atmospheric Boundary Layer Physics and Atmospheric Chemistry, Institute of Atmospheric Physics, Chinese Academy of Sciences Beijing P. R. China; Department of Chemical and Environmental Engineering, Faculty of Engineering, University of Nottingham, University Park Nottingham NG7 2RD UK; School of Environmental Science and Engineering, Nanjing University of Information Science and Technology Nanjing P. R. China; School of Public Health, University of Memphis Memphis TN USA; College of Chemistry and Materials Engineering, Wenzhou University Wenzhou P. R. China wang.chengjun@yahoo.com; Queensland Alliance for Environmental Health Sciences, The University of Queensland Brisbane Australia

## Abstract

Three monosaccharide anhydrides (MAs: levoglucosan, mannosan, and galactosan) and sugar alcohols (arabitol and mannitol) are widely used as organic tracers for source identification of aerosols emitted from biomass burning and fungal spores, respectively. In the past, these two types of organic tracer have been measured separately or conjointly using different analytical techniques, with which a number of disadvantages have been experienced during the application to environmental aerosol samples, including organic solvent involved extraction, time-consuming derivatization, or poor separation efficiency due to overlapping peaks, *etc.* Hence, in this study a more environment-friendly, effective and integrated extraction and analytical method has been developed for simultaneous determination of the above mentioned organic tracers in the same aerosol sample using ultrasonication and high performance liquid chromatography with tandem mass spectrometry (HPLC-MS/MS). The ultrasonication assisted extraction process using ultrapure water can achieve satisfactory recoveries in the range of 100.3 ± 1.3% to 108.4 ± 1.6% for these tracers. All the parameters related to LC and MS/MS have been optimized to ensure good identification and pronounced intensity for each compound. A series of rigorous validation steps have been conducted. This newly developed analytical method using ultrasonication and HPLC-MS/MS has been successfully applied to environmental aerosol samples of different pollution levels for the simultaneous measurement of the above mentioned five organic tracers from biomass burning and fungal spores.

## Abstract 2

Five organic tracers in fine aerosols can be simultaneously analysed by coupling ultrasonication and HPLC-MS/MS without a derivatization process.

## Graphical abstract

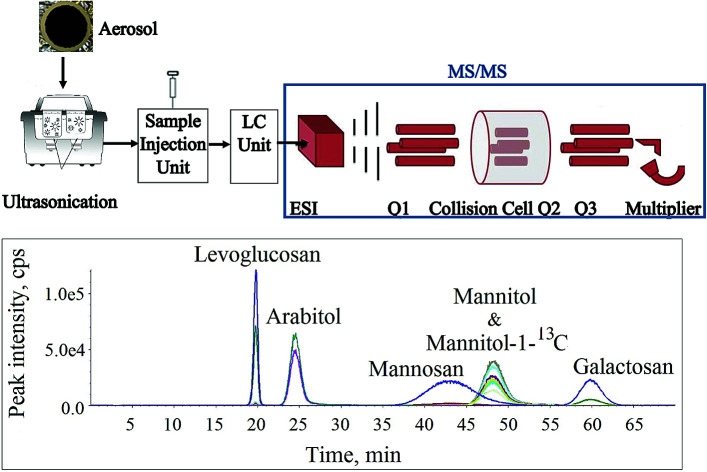

## Introduction

Biomass burning is a major contributor to the particulate matter and trace gases found in the atmosphere.^1–3^ In addition, biomass burning can also cause the release of fungal spores into the atmosphere, which may contribute to regional air pollution problems through mid- or long-range transboundary transport and pose adverse health effects such as allergic reactions.^4–8^ Biomass burning was also reported as one of the largest contributors to airborne carbonaceous aerosols.^9–12^ Particles emitted from biomass burning can act as cloud condensation nuclei in the atmosphere, change the microphysical and radiative properties and significantly affect the global climate.^13,14^ Hence, it is important to understand the characteristics of biomass burning related particles.

Water-soluble potassium was proposed as a tracer for biomass burning,^10,15,16^ but this tracer is not really unique due to its other potential sources like land or marine origins.^17,18^ Some studies calculated non-sea-salt non-dust K^+^ by eliminating these sources,^19,20^ but this correction approach is based on an assumption that no variability existed among different locations and seasons, which is clearly not applicable in all cases.^21,22^ Monosaccharide anhydrides (MAs) including levoglucosan (levo, 1, 6-anhydro-β-d-glucopyranose) and its isomers mannosan (manno, 1,6-anhydro-β-d-mannopyranose) and galactosan (gala, 1,6-anhydro-β-d-galactopyranose) are generated from the incomplete combustion or thermal alteration of cellulose and hemicellulose components of biomass under high temperature (>300 °C).^23,24^ These anhydrous sugars, especially levoglucosan, are widely employed as reliable biomass burning tracers because they are highly source specific and relatively stable in the atmosphere.^25–27^

To investigate airborne fungal spores, the traditional method based on the colony forming units assay are being used by the researchers. However, this culture method is unable to provide media in fulfilling all specific growth requirements of diverse fungal species.^28^ Microscopic counting is also widely used to quantify fungal spores.^29,30^ Nevertheless, this method can only be performed on Teflon or polycarbonate filters instead of fibrous filter media (quartz or glass fiber filters), which are commonly used for sampling to investigate the chemical composition of aerosols.^31^ Therefore, the application of molecular tracer method has been introduced to simplify sampling and allow different components of particles being determined on the same filter. It has been reported that the steroid ergosterol is considered as a marker for fungi; besides, sugar alcohols including arabitol (ara) and mannitol (manni) constitute energy reserve material in fungi and act as cell reinforcements against stressful conditions, which have also been widely employed as biomarkers.^32–36^ However, as for the estimation of fungal spore concentration in the ambient atmosphere, Filippo *et al.* (2013) pointed out that ergosterol proved to be the only reliable biomarker for fungi in their study and it should be cautious to apply mannitol and arabitol to quantify fungal contribution to the atmospheric particulate mass as other sources also release the latter two polyols, such as algae, bacteria and lower plants.^37^ On the other hand, arabitol and mannitol are produced in large amounts by many fungi and have shown to be mostly associated with fungal spores, particularly in the continental aerosols.^28,38,39^ Therefore, both mannitol and arabitol should still be applicable as biomarkers to qualitatively identify the possible inputs from fungi to the atmospheric particulate matter.^31,38,40,41^

In recent years, a lot of techniques have been developed to quantify these tracers separately or conjointly (Table 1). The most common technique to analyse MAs is gas chromatography – mass spectrometry (GC-MS) after derivatization using various silylating agents.^42,43^ Derivatization can increase the volatility and thermal stability, and improve chromatographic properties of the original polar compounds after such a conversion.^43^ The limit of detection (LOD) of levoglucosan was reported to be 130 μg L^−1^ by GC-MS.^44^ Similar to the analysis of MAs, GC-MS and gas chromatography – flame ionization detector (GC-FID) were also applied to analyse sugar alcohols after derivatization.^31,44^ Urban *et al.* (2014) applied GC-MS to analyze these tracers conjointly.^45^ However, their study lacked the discussion regarding tracer identification and separation, and this method also requires complicated derivatization as the pre-treatment process before the GC-MS analysis. GC-MS technique, as mentioned before, is a common analytical method for the analysis of these tracers, yet it is time-consuming and also may cause the analyte loss due to the derivatization process;^46–48^ though good LODs can be achieved in some studies, they failed to completely separate these tracers, which could cause bias to the final results without using tandem mass spectrometer for further peak identification.^49–52^ A number of analytical methods of MAs without derivatization have been developed in the past decade. In the study of Yttri *et al.* (2011), they applied High Performance Liquid Chromatography with High Resolution Mass Spectrometry Time-of-Flight (HPLC/HRMS-TOF) to analyze these five tracers,^53^ but these tracers were not analyzed simultaneously as different columns were used to analyze MAs and sugar alcohols, and the extraction procedure of MAs and sugar alcohols were not the same. Particle-into-liquid sampler – high-performance anion-exchange chromatography – mass spectrometry (PILS-HPAEC-MS) is a fast online determination equipment for analysing levoglucosan, the LOD of which is approximately 5–10 μg L^−1^, but its concentration is often underestimated for about 20%.^54^ Ion chromatography coupled with pulsed amperometric detector (IC-PAD) achieved the LOD of levoglucosan being only 60 μg L^−1^ (200 ng m^−3^).^55^ High-performance liquid chromatography – pulsed amperometric detector (HPLC-PAD) achieved the LODs for the above mentioned three MAs and two sugar alcohols in the range of 10–30 μg L^−1^, but a serious overlap was found between arabitol and levoglucosan.^50^ Better LODs were obtained by high-performance anion-exchange chromatography – mass spectrometry (HPAEC-MS) with 2 μg L^−1^, 1 μg L^−1^, and 1 μg L^−1^ for levoglucosan, mannosan and galactosan, respectively, in spite of small overlap observed for levoglucosan and mannosan.^49^ These five tracers were also simultaneously measured by high-performance anion-exchange chromatography – pulsed amperometric detector (HPAEC-PAD) with good LODs, but a small overlap between levoglucosan and arabitol was found.^56^ Several studies applied liquid chromatography coupled with mass spectrometry to investigate levoglucosan, mannosan and galactosan, but unsatisfactory separation always occurred.^51,52,57^ Piot *et al.* (2012) applied HPLC-MS/MS to successfully separate and determine the three MAs, however, they used sodium hydroxide as the eluent which would possibly block the electrode due to crystallization effect of this alkaline solution under high temperature at the ion source and cause non-uniform spray; besides, the LODs of MAs were 30 μg L^−1^, LOQs were 100 μg L^−1^, which were not so applicable to those less-polluted aerosol samples.^58^ HPLC-MS/MS has also been applied to analyse fungal spore tracers like arabitol and mannitol using accelerated solvent extraction with pure ethanol, the LOD of which was however not reported.^41^

**Table 1:** Comparison of analytical methods for determining aerosol sugars and sugar alcohols

Technique	Column	Eluent	Sample	Compounds	LOD (μg L^−1^)	Remark	Reference
GC-MS	DB5-MS capillary column (30 m × 0.25 mm, 0.25 μm film thickness, Agilent)	—	Soils and sediments	Levo	130	Derivatization required	44
GC-MS	DB5-MS capillary column (30 m × 0.25 mm, 0.25 μm film thickness, Agilent)	—	PM_10_	Levo, ara, manni	n.a.	Derivatization required	46
GC-MS & GC-FID	CP Sil 8CB-slow bleed capillary column (95% dimethyl, 5% phenyl polysiloxane; 30 m × 0.25 mm, 0.25 μm film thickness, Chrompack)	—	PM_10_	Levo, manno, gala	n.a.	Derivatization required; low recovery (60%)	47
GC-MS	DB5-MS capillary column (30 m × 0.25 mm, 0.25 μm film thickness, Agilent)	—	PM_10_	Levo, manno, gala	n.a.	Derivatization required; recovery of galactosan and mannosan was only 70%	48
GC-FID	Deactivated silica precolumn (0.25 mm × 2 m) and CP Sil 8CB capillary column (30 m × 0.25 mm, 0.25 μm film thickness, Chrompack)	—	PM_10_	Ara, manni	n.a.	Derivatization required	31
HPAEC-MS	CarboPac PA10 guard column (50 mm × 2 mm, Dionex) and CarboPac PA10 analytical column (250 mm × 2 mm, Dionex)	Potassium hydroxide	PM_1_	Levo, manno, gala	1–2	Small overlap of levoglucosan and mannosan; no derivatization, good LOD, repeatability and recovery	49
PILS-HPAEC-MS	—	—	PM_1_	Levo	5–10	Fast online analysis; no derivatization; underestimation of levoglucosan concentration	54
HPLC-PAD	Carbopac PA-1 guard column (50 mm × 4 mm, Dionex), and Carbopac PA-1 anion-exchange analytical column (250 mm × 4 mm, Dionex)	Sodium hydroxide	PM_10_	Levo, manno, gala, ara, manni	10–30	Serious overlap of levoglucosan and arabitol; no derivatization, good repeatability and recovery	50
HPAEC-PAD	Carbopac MA1 guard column (50 mm × 4 mm, Dionex), and Carbopac MA1 anion-exchange analytical column (250 mm × 4 mm, Dionex)	Sodium hydroxide	PM_10_	Levo, manno, gala, ara, manni	6 (levo), 4 (manno), 10 (gala), 2 (ara), 5 (manni)	Small overlap of levoglucosan and arabitol; no derivatization, good reproducibility	56
HILIC/ESI-MS/MS	Ascentis Express OH5 column (Supelco, 150 × 2.1 mm, 5 μm, Bellefone)	Water/acetonitrile 5/95 (v/v)	PM_10_	Levo, manno, gala	10.8 (levo), 18 (manno and gala)	Evaporation required for sample preparation; organic solvent required; mannosan and galactosan are completely overlapped	51
HPLC-ESI/MS	C18 column (4.6 mm × 150 mm, 5 μm, Agilent)	Methanol	Ice-core samples	Levo	10	Isomers have influence on the concentration of levoglucosan	57
HPLC/ESI-MS/MS	C18 Synergy Hydro column (50 mm × 2.1 mm, 4 μm particle size, Phenomenex)	Methanol	Ice-core samples	Levo	0.003	Fast analysis within 4 minutes; loss of chromatographic separation between levoglucosan, mannosan, and galactosan; poor repeatability (20–50%, RSD)	52
HPLC-MS/MS	Carbopac PA-1 guard column (50 mm × 4 mm, Dionex), and Carbopac PA-1 anion-exchange analytical column (250 mm × 4 mm, Dionex)	Sodium hydroxide	PM_10_ and PM_2.5_	Levo, manno, gala	30	Crystallization of eluent sodium hydroxide at the ion source; no derivatization, good separation within 6 minutes	58
HPLC-MS/MS	Carbopac MA1 guard column (50 mm × 4 mm, Dionex), and Carbopac MA1 anion-exchange analytical column (250 mm × 4 mm, Dionex)	Ammonium hydroxide	PM_2.5_	Levo, manno, gala, ara, manni	1.1–3.8	No derivatization, good LOD, repeatability, recovery and separation	This study

Hence, it has been challenging to analyze the abovementioned five organic tracers simultaneously in the same aerosol sample with pronounced intensities. To fill such a gap, ultrasonication and high performance liquid chromatography with tandem mass spectrometry (HPLC-MS/MS) were to be employed in this study and the primary objective was to develop an organic solvent free extraction process and a simultaneous analytical method with good separation, high mass selectivity and sensitivity for the identification and quantification of target compounds including three primary biomass burning tracers (levoglucosan, mannosan, and galactosan), and two fungal spore tracers (arabitol, and mannitol).

## Experimental

### Instrument, chemicals and materials

**Fig. 1:**
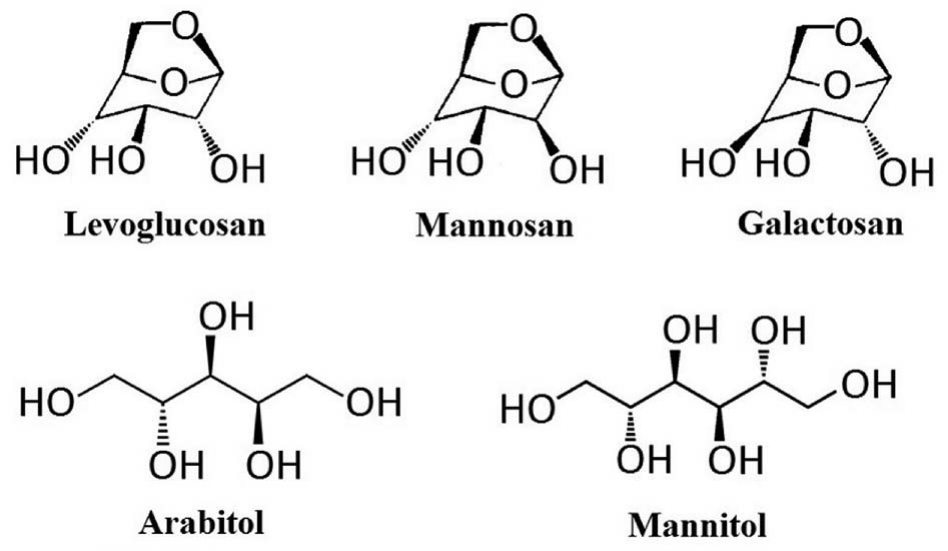
Chemical structures of levoglucosan, mannosan, galactosan, arabitol and mannitol.

The instrument applied in this study was High Performance Liquid Chromatograph (Shimadzu 30A) – Tandem Mass Spectrometry (ABSciex 3200 Q trap) (HPLC-MS/MS) equipped with Electrospray Ionization (ESI). The separation of target compounds was achieved by an anion-exchange analytical column (Dionex, Carbopac MA1, 250 mm × 4 mm) and a guard column (Dionex, Carbopac MA1, 50 mm × 4 mm). These columns were chosen due to their ability for the separation of both monosaccharides and sugar alcohols. Ammonium hydroxide (NH_3_·H_2_O, 25% NH_3_ in H_2_O, HPLC level, Sigma-Aldrich) was applied as the eluent for separation, the concentration of which could be changed through the proportion alternation of mobile phases in two pumps: pump A (ammonium hydroxide) and pump B (ultrapure water). Authentic standards applied in this study include levoglucosan (99.0%, CAS 498-07-7, Sigma-Aldrich, Shanghai, China), mannosan (98.0%, CAS 14168-65-1, Sigma-Aldrich, Shanghai, China), galactosan (97.0%, CAS 644-76-8, BOC Sciences, USA), arabitol (99.9%, CAS 488-82-4, Sigma-Aldrich, Shanghai, China) and mannitol (99.0%, CAS 69-65-8, Sigma-Aldrich, Shanghai, China), and their chemical structure are presented in Fig. 1. The internal calibration standard used was C-13 labelled mannitol (mannitol-1-^13^C, 99.0%, CAS 132202-29-0, J&K, Shanghai, China). Since all target compounds are highly water soluble, and the columns selected were not compatible with organic solvents,^59^ all solutions in this study were prepared with 18.2 Ω ultrapure water. This ultrapure water was ultrasonically degassed before preparation of ammonium hydroxide solution in order to reduce air bubbles in the elution before entering LC system,^60^ and minimize the effect of dissolved CO_2_, which could disturb chromatographic performance through the formation of carbonate.^17,61–63^

### Standard solution preparation

1000 mg L^−1^ stock solutions of levoglucosan, mannosan, galactosan and 100 mg L^−1^ stock solutions of arabitol, mannitol and mannitol-1-^13^C were prepared in ultrapure water. Working solutions (10 μg L^−1^ to 10 mg L^−1^) and external calibration standard solutions of these compounds were freshly prepared prior to analysis by diluting above-mentioned stock solutions with ultrapure water. The mixed calibration standard solutions were made by combining the aliquots of each compound stock solution and diluting with ultrapure water, resulting in mixed standards at the levels of 0.01, 0.02, 0.05, 0.1, 0.2, 0.5 and 1 mg L^−1^. Internal calibration standard mannitol-1-^13^C was added into the hybrid standards before diluting with ultrapure water to give final concentration of 1 μg mL^−1^ mannitol-1-^13^C in all standards. All solutions were stored in refrigerator at 4 °C until analysis. The linearity of calibration curves of each analyte was investigated and regularly checked throughout the entire analysis.^64^ Quality control (QC) samples were independently prepared as the calibration standards to give three levels of concentration, including low, medium and high concentrations: 20, 200, 1000 ng mL^−1^.

### Sample collection and extraction

PM_2.5_ samples were captured on 90 mm quartz fiber filters (QMA, Whatman, UK) by a medium volume PM_2.5_ samplers (model: TH-150CIII, Tianhong Instrument CO., Ltd. Wuhan, China), operating for 24 hours at a flow rate of 80 L min^−1^ at the atmospheric observation station (15 m above the ground level) in University of Nottingham Ningbo China in December 2014, January, June and July 2015. After collection, aerosol samples were wrapped in aluminium foil, sealed in polyethylene bags and stored at −18 °C until analysis. Prior to any usage, all fresh filters were pre-baked in a muffle furnace at 550 °C for 5 hours to remove any organic compounds. Before gravimetric measurement, filters were equilibrated at constant temperature (22 °C ± 1 °C) and relative humidity (30% ± 5%) for 24 h. Then the PM_2.5_ mass on 90 mm quartz fiber filters were measured by an ultra-microbalance (model: SE2-F, Sartorius, precision 0.1 μg) in the same micro-balance room.

To extract samples, one-eighth of the 90 mm filters were extracted ultrasonically by 4 mL ultrapure water for 45 minutes under room temperature, and the extracts were then filtered with 0.25 μm membrane filters and stored at 4 °C until HPLC-MS/MS analysis within one week.

## Results and discussion

### Method development

The steps in developing the HPLC-MS/MS analytical method are summarized as follows: (1) use tandem mass spectrometry (MS/MS) and manually adjust the ion source and compound parameters to identify the representative parent ions and product ions of each compound; (2) combine HPLC with MS/MS, apply the MS parameters acquired from previous step and optimize LC parameters (eluent concentration, eluent flow rate, gradient flow and column oven temperature) to achieve the best separation and highest peak intensity of representative parent with daughter (product) ion pairs in the multiple reaction monitoring (MRM) mode; (3) continue to use HPLC-MS/MS and apply above optimized LC parameters, use flow injection analysis (FIA) to obtain more precise MS parameters of interest. The optimized LC parameters in step (2) and MS parameters in step (3) would comprise the final HPLC-MS/MS method.

### Identification of representative parent and product ions

5 mg L^−1^ of levoglucosan, mannosan, galactosan, arabitol, mannitol and mannitol-1-^13^C were introduced to the mass spectrometry separately to find out their representative parent ions and product ions. MS parameters include ion source parameters, and compound parameters were manually adjusted to obtain good parent ion peak intensity and the best Collision Induced Dissociation (CID) efficiency with selective current of product ions. The mass charge ratio (*m*/*z*) of each ion is accurate to 0.1 amu (atomic mass units). Individually, levoglucosan, mannosan and galactosan have the same parent ion *m*/*z* 160.9, and those for arabitol, mannitol and mannitol-1-^13^C were *m*/*z* 151.0, *m*/*z* 181.0 and *m*/*z* 182.0, respectively. The parent and product ions for levoglucosan, mannosan, galactosan, arabitol, mannitol and mannitol-1-^13^C were presented in Table 2. The MRM acquisition mode was applied to acquire two or more diagnostic product ions from the chosen parent ions (as mentioned above) to obtain high selectivity and sensitivity. The data were collected in negative ion mode with a 200 ms dwell time/transition. The injection volume was 10 μL. The parent ions were selected in the first quadrupole (Q1) in MS and the product ions of interest were selected in the third quadrupole (Q3) in MS.

**Table 2:** Analytical parameters of each organic tracer in MRM mode of HPLC-MS/MS

Tracers	RT^a^ (min)	Parent ion	Product ion	Declustering potential DP (V)	Entrance potential EP (V)	Collision cell entrance potential CEP (V)	Collision energy CE (V)	Collision cell exit potential CXP (V)
Levo	19.8	**160.9** b	101.0*	−45	−2.2	−9	−15	−1.7
		160.9	113.0	−42	−2.0	−6	−13	−2.2
Manno	43.0	**160.9**	**101.0**	−45	−2.2	−9	−15	−1.7
		160.9	129.0	−38	−3.2	−6	−13	−2.2
Gala	59.9	**160.9**	**101.0**	−45	−2.2	−9	−15	−1.7
		160.9	113.0	−42	−2.3	−12	−13	−1.7
Ara	24.5	**151.0**	**71.0**	−32	−2.5	−10	−25	−1.2
		151.0	59.0	−30	−3.0	−10	−28	−1.0
Manni	48.3	**181.0**	**89.1**	−32	−2.8	−15	−20	−1.7
		181.0	71.0	−32	−2.2	−8	−30	−1.2

a Retention time.

b The ion pairs highlighted in bold were used for the quantification of each compound.

### Optimization of LC parameters

Anion-exchange analytical columns were used for separation of these compounds. Given its compatibility requirement, hydroxide eluent is needed as a mobile phase. Sodium hydroxide, as a non-volatile compound, will crystallize under high temperature and very possibly block the ion source to cause non-uniform spray, which deems not suitable as an eluent for LC-MS unless it is equipped with a desalting unit upstream. Hence, in this study, the suitability of ammonium hydroxide was explored. The preliminary tests showed that the gradient elution of ammonium hydroxide has little impact on the peak separation, thus the optimization of LC parameters is based on column oven temperature, eluent flow rate and concentration.

#### (1) Column oven temperature

According to single variable principle, the column oven temperature was the only parameter to be optimized in this step. MS parameters were not changed after the acquisition of parent and product ions from previous step. The applied eluent was 0.001% NH_3_·H_2_O with isocratic flow rate at 0.4 mL min^−1^. The applicable temperature range of the columns is 4–50 °C;^61^ to ensure consistent good performance of the columns, the temperature should not be higher than 90% of its maximum. Therefore, the column oven temperature was optimized at 25, 30, 35, 40 and 45 °C. When the temperature was lower than 35 °C, the separation of mannosan and galactosan was not satisfactory. As the parent-product ion pair (160.9–101.0) selected for both was the same, it was impossible for identification and quantification when they were overlapped. Therefore, only 40 °C and 45 °C were considered to achieve this purpose. Regarding the peak intensity, levoglucosan was observed with highest intensity under 40 °C, while others were with highest intensities at 45 °C. The ambient concentrations of mannosan, galactosan, arabitol and mannitol are usually lower than levoglucosan. Therefore, to make sure they all can be detected at trace levels in the same aerosol sample, 45 °C was finally selected and applied in this study.

#### (2) Eluent flow rate

It has been well established that operating ESI at low flow rates improves ionization efficiency while the ion transmittance current at the ESI interface could increase at higher flow rates to some extent.^65–67^ Though these MAs are stereoisomers with similar physical and chemical properties, it is still possible for them to respond to the change of flow rates slightly differently in terms of both ionization and transmission efficiencies, which may lead to the maximum peak intensity of each individual analyte at different optimum flow rate. Hence the flow rate optimization was necessary in this study.

The flow rate applicable to the columns is in the range of 0.2–0.5 mL min^−1^.^61^ However, during the optimization process, the column pressure occasionally reached its limit (2000 psi) when the 0.45 mL min^−1^ was applied as the flow rate. Hence, the flow rates for optimization were set as 0.2, 0.25, 0.3, 0.35 and 0.4 mL min^−1^. At 0.35 and 0.4 mL min^−1^, the intensities of most organic tracers were higher than those acquired at other flow rates; eventually 0.4 mL min^−1^ was selected as optimum flow rate, which is also beneficial to the reduction of analysis time per sample while the good separation is still achieved.

#### (3) Eluent concentration

**Fig. 2:**
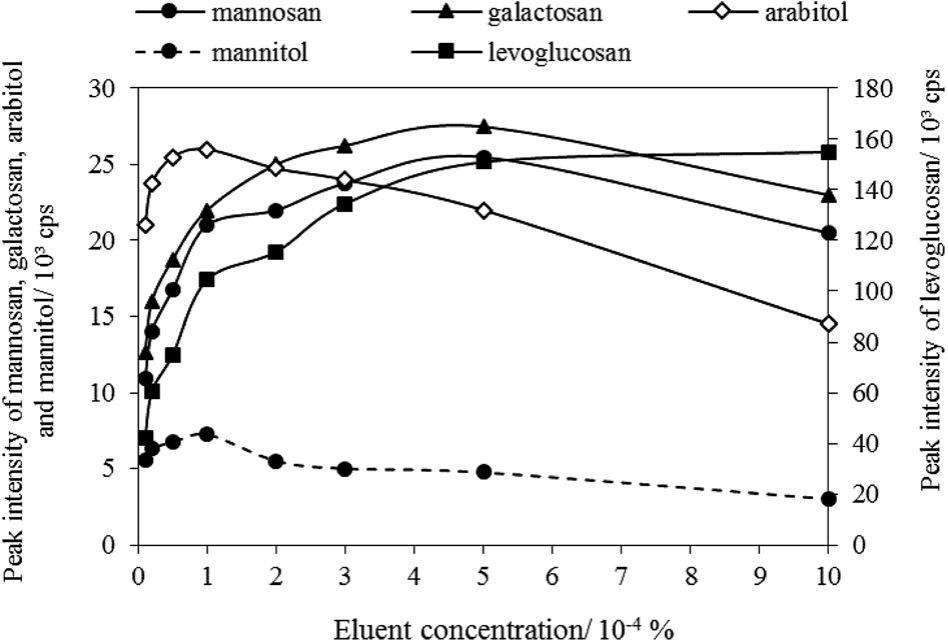
Eluent concentration optimization.

In this step, the column oven temperature and eluent flow rate were set as 45 °C and 0.4 mL min^−1^, respectively. The optimization results regarding eluent concentration was plotted as shown in Fig. 2. It shows that levoglucosan, with the highest intensity among all tracers, was highly affected by the change of ammonium hydroxide solution, and its intensity increased as the eluent concentration increased in the range of 0.00001–0.001%. The peak intensities of mannosan and galactosan were the highest when the eluent concentration is 0.0005%. However, arabitol and mannitol were observed with the highest peak intensity when eluent concentration was 0.0001%. Because the peak intensities of arabitol and mannitol were lower than those of the three MAs, and the ambient concentrations of arabitol and mannitol were generally lower than MAs, consistent with the published studies.^50,68–70^ Therefore, to ensure all five tracers can be detectable in the same aerosol sample of low levels, the optimum ammonium hydroxide concentration was selected as 0.0001% (approximately 0.0528 mmol L^−1^).

### Optimization of MS parameters through FIA analysis

After the optimization of LC conditions, corresponding MS parameters need to be adjusted as well to achieve the best peak intensity. Flow injection analysis (FIA) was applied in this study to automatically optimize the MS parameters of interest, under which the highest peak intensities could be achieved eventually. The ion sources parameters after optimization are listed as follows: curtain gas (CUR), 46.0 psi; ion spray voltage (IS), −3800 V; source temperature (TEM), 650 °C; Ion source gas 1/nebulizer gas (gas 1), 38 psi; ion source gas 2/auxiliary gas (gas 2), 80 psi. Detailed information of the optimized method is presented in Table 2, including the retention times (RT), the parent and product ions and the compound parameters of the tracers.

### Method performance

**Fig. 3:**
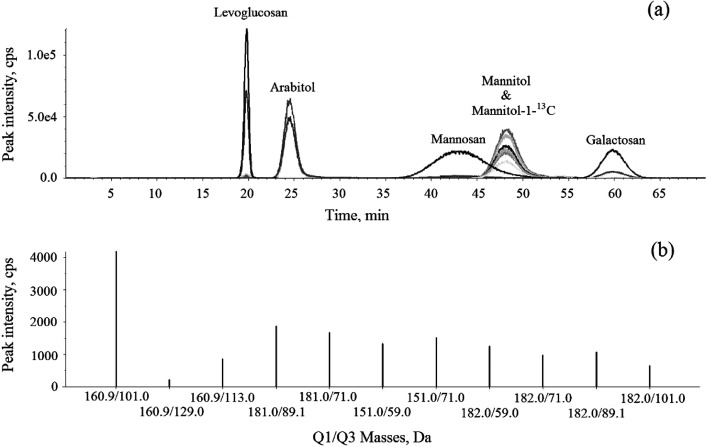
The HPLC-MS/MS (a) MRM chromatogram and (b) Q1/Q3 mass spectrometry on selective *m*/*z* of levoglucosan, mannosan, galactosan, arabitol, mannitol and mannitol-1-^13^C in 1 mg L^−1^ standard solution.

The HPLC-MS/MS MRM chromatogram and Q1/Q3 (parent-product ion pairs) mass spectrometry on selective *m*/*z* of levoglucosan, mannosan, galactosan, arabitol, mannitol and mannitol-1-^13^C in 1 mg L^−1^ standard solution are shown in Fig. 3. Each ion pair was corresponding to a line in the chromatogram and could be extracted separately. The ones with the highest intensity of each compound were chosen for their quantification respectively, shown in bold in Table 2. The resulting chromatogram exhibit good separations for levoglucosan, arabitol and galactosan, but the peaks of mannosan and mannitol were partially overlapped in the figure, moreover, mannitol and mannitol-1-^13^C shared the same retention time. However, as mannosan, mannitol and mannitol-1-^13^C have completely different representative parent-product ion pairs, and each ion pair can be extracted from the chromatogram individually in MRM mode, therefore their quantifications are not affected through the integration of respective peak areas, which demonstrates the advantageous selectivity of tandem mass spectrometry.

### Method validation

Validation of this method was conducted in terms of the following aspects: linearity, sensitivity, selectivity, specificity, accuracy and precision, extraction recovery, matrix effect, reproducibility and stability. The LOD, calibration curves, extraction recovery and reproducibility of the five above mentioned organic tracers and internal standard mannitol-1-^13^C are presented in Table 3.

**Table 3:** The LOD, calibration curve, recovery and reproducibility of five tracers by HPLC-MS/MS in MRM mode

Compounds	LOD		Calibration curve		Extraction^b^ recovery%	Reproducibility	
	Injected volume (μL)	Extract conc. (μg L^−1^)	Conc. range (mg L^−1^)	*R*		Area^c^ RSD%	Area^d^ RSD%
Levo	10	1.1	0.01–1	0.9992	108.4 ± 1.6	2.0	0.6
Ara	10	3.8	0.01–1	0.9998	101.3 ± 2.3	1.4	0.4
Manno	10	2.3	0.01–1	0.9999	104.7 ± 1.8	1.9	7.7
Manni	10	3.3	0.01–1	0.9995	100.3 ± 1.3	2.2	1.8
Mannitol-1-^13^C	10	N/A^a^	0.01–1	0.9996	101.3 ± 1.8	1.6	N/A
Gala	10	1.2	0.01–1	0.9999	106.5 ± 1.9	1.6	5.1

a Not available.

b Recovery of spiked standards (mean ± SD).

c Standard solutions, *n* = 5.

d Atmospheric aerosol samples, *n* = 4.

### Linearity, sensitivity, selectivity and specificity

**Fig. 4:**
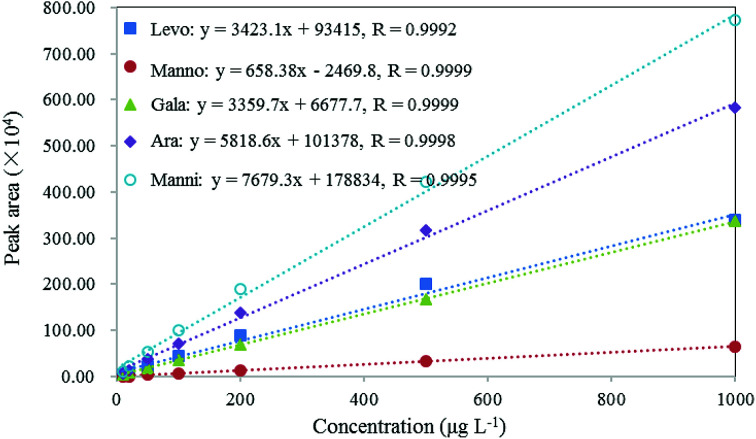
Calibration curves, correlation coefficient (*R*) of levoglucosan, mannosan, galactosan, arabitol and mannitol.

As shown in Fig. 4, the calibration curves of these compounds demonstrated excellent linearity with linear correlation coefficients (*R*) > 0.999 in the concentration range of 0.01–1 mg L^−1^. The LOD can be used to evaluate the sensitivity of the method, which was calculated as the concentration that corresponds to three times the standard deviation of the peak areas produced by filter blanks spiked with certain amount of standards.^71–75^ In this study, to evaluate LODs, 20 μL of 2 mg L^−1^ standard solutions were spiked on blank filters to result in 10 μg L^−1^ of the standards in extracts. The LODs of these compounds range from 1.1 μg L^−1^ for levoglucosan to 3.8 μg L^−1^ for arabitol, as presented in Table 3. These results indicate that this method is sufficiently sensitive for the analysis of target biomarkers in aerosol samples.

**Fig. 5:**
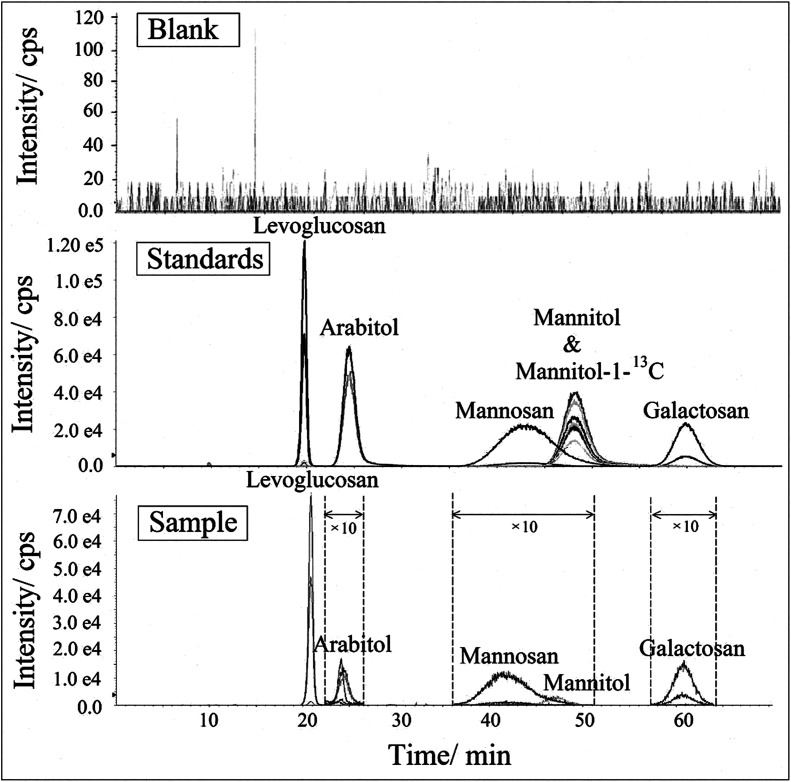
The HPLC-MSMS chromatogram of levoglucosan, mannosan, galactosan, arabitol, mannitol and mannitol-1-^13^C on selective *m*/*z* in standard and sample extracts (including both blank and environmental samples).

All analytes were identified by both each individual's retention time and specific MRM transitions. MRM in LC-MS/MS is very powerful to identify and specify target compounds in complex matrices,^76^ as only specific parent and product ions (parent mass → fragment mass) are selected to be examined. The selectivity of this method was evaluated by comparing the chromatograms acquired from extracts of field blanks and environmental aerosol samples and prepared standard solutions,^77,78^ as shown in Fig. 5. Because it is difficult to recognize interference signals from matrices, Hess *et al.* (2018) recommended that it is better to analyse more than 20 matrices to evaluate possible selectivity problems for endogenous substances.^79^ In this study, we analysed more than 40 blanks, which were collected from different representative areas, such as rural, urban and suburban areas, on seasonal basis (unpublished data from our other study). A typical chromatogram from these field blank samples was chosen and presented in Fig. 5. For better presentation, the peak intensities of arabitol, mannosan, mannitol and galactosan in the sample chromatograms were enlarged by 10 times due to their relatively lower concentrations in aerosol samples. No interference was observed at the retention times of these biomarkers in field blank. Additionally, the peak properties such as retention time and peak shape of these compounds in aerosol sample were observed with high consistence with those in standard solution, which indicates reliable selectivity and specificity of this newly developed method.

### Accuracy and precision

The inter- and intra-day accuracy and precision were estimated by analyzing three replicates at low (20 ng mL^−1^), medium (200 ng mL^−1^) and high (1000 ng mL^−1^) quality control (QC) levels, as summarized in Table 4. The intra-day accuracy and precision data was obtained from measurements within one day while the inter-day data was determined on three different days. Accuracy was defined as the percentage of the measured concentration to the nominal concentration of QC samples. Precision is evaluated through the calculation of the coefficient of variation (CV (%) = standard deviation/mean × 100%). The intra-day precision was <10% for all analytes in low and medium QCs, and high QCs were observed with excellent intra-day precision (<5%) for all analytes. The inter-day precision was <15% at all QC levels. For intra-day accuracy, low QCs exhibited accuracy <15% for most tracers except for mannosan (119%), the medium and high QCs demonstrated all accuracy within 5%. The inter-day accuracy was within 10% for all analytes. These results indicate that the present method is accurate and precise for the determination of levoglucosan, mannosan, galactosan, arabitol and mannitol in aerosol samples.

**Table 4:** Inter- and intra-day precision and accuracy of QC samples (*n* = 3)

Compounds	Concentration (ng mL^−1^)	Inter-day			Intra-day		
		20	200	1000	20	200	1000
Levoglucosan	Mean ± SD	19.7 ± 0.1	197.6 ± 6.3	1009.7 ± 40.0	20.8 ± 0.4	206.1 ± 14.2	1047.1 ± 43.0
	CV (%)	0.5%	3.2%	4.0%	2.0%	6.9%	4.1%
	Accuracy (%)	98.5%	98.8%	110.0%	104.0%	103.1%	104.7%
Mannosan	Mean ± SD	21.4 ± 0.8	199.7 ± 1.9	996.4 ± 22.8	23.8 ± 2.0	207.0 ± 11.7	1044.1 ± 48.6
	CV (%)	3.7%	0.9%	2.3%	8.3%	5.6%	4.7%
	Accuracy (%)	107.0%	99.9%	99.6%	119.0%	103.5%	104.4%
Galactosan	Mean ± SD	21.4 ± 0.5	200.7 ± 1.8	997.7 ± 17.8	22.8 ± 0.8	210.0 ± 13.2	1035.1 ± 42.4
	CV (%)	2.1%	0.9%	1.8%	3.6%	6.3%	4.1%
	Accuracy (%)	107.0%	100.4%	99.8%	114.0%	105.0%	103.5%
Arabitol	Mean ± SD	19.3 ± 2.0	194.4 ± 12.1	978.7 ± 12.3	21.0 ± 0.4	203.6 ± 7.5	1027.5 ± 33.6
	CV (%)	10.6%	6.2%	5.4%	2.0%	3.7%	3.3%
	Accuracy (%)	96.5%	97.2%	97.9%	105.0%	101.5%	102.8%
Mannitol	Mean ± SD	18.6 ± 2.7	190.7 ± 20.5	978.1 ± 24.9	19.4 ± 1.1	198.9 ± 11.1	1021.7 ± 26.7
	CV (%)	14.7%	10.8%	7.8%	5.6%	5.6%	2.6%
	Accuracy (%)	93.0%	95.4%	97.8%	97.0%	99.5%	102.2%

### Extraction recovery

The extraction recovery of the five target organic tracers were then tested by spiking a certain small amount of stock solutions onto the pre-baked blank filters. The spiked filters were dried at constant temperature (22 °C ± 1 °C) and relative humidity (30% ± 5%) for 24 h and then extracted in the same way as previously described in Section 2.3, in which the prepared concentrations of 1 mg L^−1^ for each compound should be produced in final extracts. The extraction recovery of levoglucosan, mannosan, galactosan, arabitol and mannitol were found to be 108.4 (±1.6)%, 104.7 (±1.8)%, 106.5 (±1.9)%, 101.3 (±2.3)% and 100.3 (±1.3)%, respectively, as shown in Table 3. The concentrations of these compounds on blank filters were all below detection limits. The high extraction recoveries confirm that ultrapure water instead of organic solvent can be applied to extract these five organic tracers from aerosol samples by ultrasonication efficiently, indicating such an extraction process coupled with the newly developed LC-MS/MS analytical method would be more environment-friendly.

### Matrix effect

The matrix effects of sampling substrate (filter) were roughly evaluated in the following manner. The analytes of interest with the same concentration levels were added into the pre-baked filters and ultrapure water, which were then analyzed by the LC-MS/MS method and the ratio of signals generated by the analytes of interest extracted from standard-spiked filters and those form standard-spiked pure water samples.^78,80^ Three concentrations of 20, 100, 500 μg L^−1^ were tested for matrix effects, with three replicates of each level. The matrix effects of levoglucosan, mannosan, galactosan, arabitol and mannitol were 107.7 ± 1.3%, 102.9 ± 2.1%, 104.7 ± 1.6%, 103.3 ± 1.0% and 101.4 ± 1.5%, respectively, indicating the matrix effects from sampling substrate were insignificant.

### Reproducibility

Reproducibility of this method was also tested for both standard solutions and atmospheric aerosol samples. As shown in Table 3, for consecutive analyses of standard solutions (*n* = 5), the relative standard deviations (RSD%) of peak area were lower than 2.2% for all compounds. For consecutive analyses of atmospheric aerosol samples (*n* = 4), the peak area RSD% of levoglucosan, arabitol and mannitol were 0.6%, 0.4% and 1.8%, respectively. However, higher RSDs were found for mannosan (7.7%) and galactosan (5.1%) than the former three analytes in the atmospheric aerosol samples, yet in an acceptable range.^81^ This observation was comparable with that reported in the literature (three MAs average: 6.7%) in aerosol samples for high performance liquid chromatography with aerosol charge detection method.^82^ Hence, the reproducibility of this method was considered as good enough for both standard solutions and environmental aerosol samples.

### Stability

Stability of extracts was evaluated using standards spiked blank filters. As presented in Table 5, four different concentrations (10, 50, 500 and 1000 ng mL^−1^) of standards were tested for stability to validate it as a reliable method to analyse the aerosol samples within the linear range. The stability of the QC samples was tested at day 0, then stored at 4 °C and re-measured at day 1 and day 7. For stability in 24 hours, each concentration of the analytes in four replicates was measured immediately after extraction and filtration and re-measured after 24 h storage at 4 °C. Then, the QC samples were stored at 4 °C again until analysis at day 7. In this study, both RSD and relative error (RE) were applied to statistically assess the extract stability. Based on the principles for analytical method validation, both RSD and RE within 15% are widely recognized and employed as an acceptance criteria for such an evaluation.^83–86^ The RE was calculated using the following formula:RE = [(measured concentration at day 1 or day 7 − measured concentration at day 0)/measured concentration at day 0] × 100%

**Table 5:** Stability of levoglucosan, mannosan, galactosan, arabitol and mannitol in standards at 4 °C (*n* = 4)

Compounds	Concentration measured at day 0 (ng mL^−1^)	24 h stability			One-week stability		
		Concentration measured at day 1 (ng mL^−1^)	RSD	RE	Concentration measured at day 7 (ng mL^−1^)	RSD	RE
Levoglucosan	10.5 ± 0.4	10.7 ± 0.4	4.1%	6.8%	10.4 ± 0.4	3.4%	4.3%
	51.4 ± 1.1	51.4 ± 1.0	1.9%	2.8%	50.7 ± 0.6	1.2%	1.4%
	516.8 ± 16.2	524.5 ± 21.8	4.2%	4.9%	510.5 ± 15.8	3.1%	2.1%
	1050.8 ± 35.4	1063.8 ± 24.4	2.3%	6.4%	1043.3 ± 34.0	3.3%	4.3%
Mannosan	10.9 ± 1.0	10.5 ± 0.6	6.1%	5.1%	10.1 ± 0.7	6.8%	1.0%
	52.8 ± 2.3	53.5 ± 2.4	4.5%	6.9%	53.1 ± 2.7	5.0%	6.2%
	516.4 ± 10.1	518.8 ± 18.4	3.5%	3.8%	507.3 ± 7.2	1.4%	1.5%
	1044.9 ± 3.4	1039.4 ± 6.9	0.7%	3.9%	1028.0 ± 20.2	2.0%	2.8%
Galactosan	10.8 ± 0.9	10.7 ± 0.7	6.7%	6.6%	10.1 ± 0.6	5.8%	1.0%
	52.9 ± 0.1	52.6 ± 0.3	0.6%	5.1%	52.3 ± 0.4	0.8%	4.5%
	517.5 ± 8.5	520.4 ± 19.4	3.7%	4.1%	508.2 ± 7.3	1.4%	1.6%
	1054.7 ± 18.9	1040.3 ± 28.3	2.7%	4.0%	1035.1 ± 34.6	3.3%	3.5%
Arabitol	11.0 ± 0.3	10.7 ± 0.6	5.6%	7.5%	9.4 ± 0.5	5.1%	−6.2%
	51.6 ± 0.7	49.9 ± 1.4	2.9%	−0.2%	49.4 ± 0.9	1.8%	−1.3%
	533.4 ± 17.7	506.9 ± 18.9	3.7%	1.4%	487.9 ± 21.5	4.4%	−2.4%
	1024.8 ± 8.2	1017.0 ± 34.5	3.4%	1.7%	976.9 ± 49.2	5.0%	−2.3%
Mannitol	10.4 ± 0.4	9.6 ± 0.6	5.9%	−4.3%	9.2 ± 0.2	2.5%	−8.2%
	50.8 ± 0.8	49.0 ± 1.5	3.1%	−2.1%	48.6 ± 1.0	2.0%	−2.9%
	504.4 ± 10.5	501.5 ± 21.1	4.2%	0.3%	487.2 ± 16.9	3.5%	−2.6%
	1012.3 ± 30.1	1008.0 ± 35.0	3.5%	0.8%	960.0 ± 69.6	7.3%	−4.0%

The RSDs of levoglucosan, mannosan, galactosan, arabitol and mannitol were in the range of 0.6–6.7% in measurements after 24 hours, and 0.8–7.3% in measurements after one week. The stability evaluation showed a slight decrease of analyte concentrations after storing at 4 °C for one week. Few negative values of REs were observed for arabitol and mannitol after a short-term storage, which was probably the result of volatilization of sugar alcohols. However, both RSDs and REs of all biomarkers at different concentration levels were all within 10% and met the abovementioned performance criteria. Hence, sample extracts can be stored for a short period of time at 4 °C (*i.e.* 7 days) prior to HPLC-MS/MS analysis without major adverse effects on the experimental results.

### Method application to the environmental aerosol samples

Levoglucosan, mannosan, galactosan, arabitol and mannitol were determined in the atmospheric aerosol samples collected in Ningbo during winter (December 2014 to January 2015) and summer periods (June–July 2015). All the daily PM_2.5_ concentrations are presented in Table 6. As benchmarked against the Chinese Ambient Air Quality Standards (CAAQS, level 2 applicable for residential, commercial, industrial and rural areas) daily PM_2.5_ threshold of 75 μg m^−3^, the winter sampling period experienced moderate to high aerosol pollution levels with an average PM_2.5_ concentration of 88.1 ± 24.3 μg m^−3^ ranging from 47.5 to 127.4 μg m^−3^, while much lower aerosol pollution was observed during the summer season with an average PM_2.5_ concentration of 27.2 ± 6.4 μg m^−3^ in the range of 17.0–36.1 μg m^−3^. Fig. 5 shows a representative chromatogram of five tracers from an aerosol sample and a mixed standard solution. As can be seen in Fig. 5, these compounds are undetectable in field blank filters. Table 6 additionally summarizes the concentrations all biomarkers of interest in aerosol samples during winter and summer seasons. To attain good quality assurance and control, for the daily analysis of one batch aerosol samples, a new calibration curve was always established at the beginning of measurement. In addition, another calibration curve was also made right after the analysis of that batch samples mentioned above to ensure the RSD% of the concentrations of all analytes in the analysed samples using two separate calibration curves were all <5%, otherwise those particular samples which did not meet such a requirement would have to be re-analysed. Furthermore, the analytical columns were equilibrated between two injections for 3 minutes, for which the same LC parameters were employed as for the sample analysis. Every two weeks, without injecting the mobile phase into MS, the columns were flushed with 200 mmol L^−1^ sodium hydroxide solution at a flow rate of 0.4 mL min^−1^ for 24 h for the removal of residues to maintain their good performance.^58,61^

**Table 6:** The concentrations of levoglucosan, mannosan, galactosan, arabitol and mannitol in PM_2.5_ samples by applying the new HPLC-MS/MS method

	Date	PM_2.5_ (μg m^−3^)	Levoglucosan (ng m^−3^)	Mannosan (ng m^−3^)	Galactosan (ng m^−3^)	Arabitol (ng m^−3^)	Mannitol (ng m^−3^)
Winter samples	2014-12-03	63.4	155.2	13.8	8.6	7.7	4.9
	2014-12-09	47.5	310.0	30.4	17.0	10.5	6.0
	2014-12-15	127.4	626.1	47.9	37.7	28.1	19.3
	2014-12-21	93.0	304.9	32.4	19.8	14.2	9.8
	2014-12-29	86.8	414.3	41.6	22.1	9.0	4.6
	2015-01-02	87.0	335.2	43.1	23.9	7.1	4.8
	2015-01-08	103.1	252.1	35.9	21.1	6.8	4.3
	2015-01-22	97.0	132.3	14.7	9.6	4.5	3.5
Summer samples	2015-06-02	36.1	45.8	3.5	2.1	8.4	8.3
	2015-06-06	28.5	18.1	1.3	1.6	7.4	7.9
	2015-06-11	25.2	114.4	9.1	3.7	11.3	14.9
	2015-06-14	17.0	50.1	5.1	2.4	7.7	7.4
	2015-06-24	23.9	34.5	2.2	1.3	8.0	12.1
	2015-07-14	34.0	24.0	1.6	1.0	8.0	9.3
	2015-07-20	21.8	55.8	2.6	2.2	7.1	7.2
	2015-07-26	30.8	76.9	4.6	2.5	9.4	12.6

The aerosol samples were extracted by standard extraction procedure as mentioned earlier in the Experimental section. The concentrations of biomarkers in sample extracts (ng mL^−1^) were converted into their corresponding concentrations in the atmosphere (ng m^−3^). The results showed a successful application of this newly developed method to identify and quantify these five biomarkers of interest at various pollution levels (PM_2.5_: 17.0–127.4 μg m^−3^). During the sampling period, levoglucosan concentrations ranged between 18.1–626.1 ng m^−3^, with average values of 316.3 ± 155.9 and 52.5 ± 31.2 ng m^−3^ during winter and summer periods, respectively. As presented in Table 7, it is in good agreement with those measured in Brno, Czech Republic and Shanghai, China,^87,88^ but higher than those obtained in Hong Kong,^89^ where less aerosol pollution is often found compared to the area in this study.^90^ The concentrations of galactosan in winter and summer periods were 20.0 ± 9.1 and 2.1 ± 0.8 ng m^−3^, which are consistent with the results reported in Belgium during winter (19.6 ng m^−3^),^47^ and Maine, USA during summer (1.1 ng m^−3^),^91^ respectively. The arabitol and mannitol in this study were 11.0 (±7.5) and 7.1 ng m^−3^ (±5.3) during winter and 8.4 (±1.4) and 10.0 ng m^−3^ (±2.9) during summer, respectively, which are lower than those obtained in PM_10_ samples of Vienna during summer,^31^ but comparable to other studies.^32,91,92^ The seasonal mean concentrations of mannosan were 32.5 ± 12.6 and 3.8 ± 2.6 ng m^−3^ during winter and summer, respectively. A study in another Chinese city – Chengdu revealed comparable levoglucosan and mannosan concentrations, but relatively higher arabitol (21.5 ng m^−3^) and mannitol (43.9 ng m^−3^) concentrations than those in this study.^35^ They explained such unexpectedly higher levels of sugar alcohols were mainly influenced by biomass burning plumes during a biomass burning season from April to May 2009. In our study, biomass burning may have also contributed to fungal spore tracers, as the maximum values of sugar alcohols coincided with the peak levels of biomass burning tracers on December 15, 2014.

**Table 7:** Literature data of the concentrations of levoglucosan, mannosan, galactosan, arabitol and mannitol (ng m^−3^) in aerosol samples

Sampling location	Samples	Sampling period	Levoglucosan	Mannosan	Galactosan	Arabitol	Mannitol	References
Brno, Czech Republic	PM_2.5_	Winter	326 ± 114	73.4 ± 23.4	34.9 ± 6.13	—	—	87
		Summer	47.1 ± 26.4	24.0 ± 2.95	18.9 ± 1.85	—	—	
Ghent, Belgium	PM_10_	Winter	477	66	19.6	—	—	47
Budapest	PM_10_	Feb–Mar 2014	387	28	16	7.5	4.7	92
Maine, USA	Aerosol (>1 μm)	Summer (Jul 2002)	54.0	7.6	1.1	5.0	5.8	91
Vienna	PM_10_	Summer	—	—	—	28	42	31
Hainan, China	PM_2.5_	Apr–May 2004	—	—	—	7.0	16.0	32
Chengdu, China	PM_2.5_	Apr–May 2009	396.5	21.9	—	21.5	43.9	35
Hong Kong, China	PM_2.5_	Winter	190	—	—	—	6.0	89
		Summer	35.2	—	—	—	3.5	
Shanghai, China	PM_2.5_	Winter	392.2	126.5	—	7.3	36.8	88
		Summer	46.5	9.2	—	14.9	172.7	
Ningbo, China	PM_2.5_	Winter	316.3 ± 155.9	32.5 ± 12.6	20.0 ± 9.1	11.0 ± 7.5	7.1 ± 5.3	This study
		Summer	52.5 ± 31.2	3.8 ± 2.6	2.1 ± 0.8	8.4 ± 1.4	10.0 ± 2.9	

## Conclusions

A pure water based ultrasonic extraction integrated with HPLC-MS/MS method has been developed in this study for the fast and simultaneous measurement of three primary biomass burning tracers (levoglucosan, mannosan, and galactosan) and two fungal spore tracers (arabitol, and mannitol). The effective identification of representative parent and product ion pairs for each tracer can be acquired using the MRM acquisition mode and their separation are also achieved based on the optimized oven temperature, eluent flow rate and concentration; in addition, the operating condition for tandem mass spectrometry has also been determined using flow injection analysis. This newly developed method has been proved simple, reliable and effective in terms of its good calibration curve linearity, sensitivity, selectivity, accuracy, precision, reproducibility and stability, which have been demonstrated in the vigorous validation section. Finally, this method has been successfully applied to the environmental fine aerosol samples collected in an eastern coastal city, Ningbo of China, to identify and quantify multiple biomass burning and fungal spore biomarkers at ng m^−3^ levels.

## Conflicts of interest

There are no conflicts to declare.

## Supplementary Material
